# Biological functions and prognostic value of RNA Binding Proteins in clear cell Renal Cell Carcinoma

**DOI:** 10.7150/jca.49175

**Published:** 2020-09-23

**Authors:** Zhenpeng Zhu, Anbang He, Lanruo Lin, Chunru Xu, Tianyu Cai, Jian Lin

**Affiliations:** 1 Department of Urology, Peking University First Hospital, Beijing 100034, China; 2 Institute of Urology, Peking University, Beijing 100034, China; 3 Capital Medical University, Beijing 100069, China

**Keywords:** clear cell renal cell carcinoma, RNA Binding Proteins, Bioinformatics analysis, Prognostic signature, Overall survival.

## Abstract

Early detection and accurate evaluation were both critical to improving the prognosis of clear cell Renal Cell Carcinoma (ccRCC) patients. More importantly, RNA Binding Proteins (RBPs) play a vital role in the tumorigenesis and progression of numerous cancers. However, the relationship between RBPs and ccRCC is still unclear. Exploring the potential biological functions of RBPs in ccRCC and establishing a prognostic signature to predict the survival probability remains meaningful. In this study, transcriptome profiling and the corresponding clinical information were obtained from the TCGA database, GEO database, and ICGC database. By using the "edgeR" R package, 200 DERBPs were found, including 128 up-regulated and 72 down-regulated RBPs. Gene Ontology (GO) and Kyoto Encyclopedia of Genes and Genomes (KEGG) enrichment analyses showed that DERBPs were mainly involved in regulating transcriptional processes and metabolism. Furthermore, there were 4 hub genes (RPS2, RPS14, RPS20, and RPLP0) were found in the PPI network, which may play vital biological roles among those DERBPs. Then we used LASSO regression to construct a prognostic signature and validated the signature in the GEO and ICGC cohort. The time-dependent receiver operating characteristic (ROC) curve showed that the signature could accurately predict the prognosis of ccRCC patients. Then we established a nomogram, and the calibration curve and ROC curve showed that the nomogram could accurately predict 1-year, 3-year, and 5-year overall survival (OS) of ccRCC patients (The AUC value: 0.871, 0.829, and 0.816). In conclusion, we constructed a 10-RBPs-based prognostic signature integrating clinical parameters to predict the prognosis of ccRCC patients. The prognostic signature based on the differentially expressed RBPs (DERBPs) might serve as promising diagnostic and prognostic biomarkers in ccRCC.

## Introduction

Renal cell carcinoma (RCC) is one of the most common and deadly cancer worldwide, with an incidence rate that is increasing 2% each year. Clear cell Renal Cell Carcinoma (ccRCC) is the major histological subtype of RCC, accounting for 70%-80% cases 1, 2. Earlier diagnoses of ccRCC could expand the life expectancy of cancer survivors and contribute to the preservation of the functions of the kidney 3, 4. Moreover, the genetic alterations behind ccRCC have been widely studied using bioinformatics analyses. The bioinformatics analyses have become one of the most effective tools for analyzing ccRCC^5^, ^6^. According to the previous studies, numerous biomarkers are correlated with prognosis or diagnosis, such as Aquaporin 9 and RURKB, which were reported recently 7, 8. However, there are still fewer biomarkers in ccRCC meaningful to personalized treatment or clinical diagnosis. Hence, due to the high morbidity of ccRCC, it is still important to explore more meaningful biomarkers for early diagnosis and personalized treatment.

RBPs were firstly identified for their critical and conserved roles in RNA binding 9. Previous studies showed that RBPs interacted with mRNA major depended on RNA-Binding Domain, such as RNA-recognition motif, K-homology domain, Zinc fingers, etc. 10. There are almost 1912 RBPs, according to recent studies 11. To our best knowledge, RBPs participated in many vital processes, including RNA splicing, translation, and stability 12-14. More importantly, the dysregulated expression of some RBPs was associated with disease occurrence, including genetic disease and cancer 15, 16. In our previous studies, we found that P4HB, as an RNA Binding Protein, was up-regulated both in mRNA and protein expression levels and correlated with poor prognosis in ccRCC patients 17. Other studies had also pointed some of RBPs were associated with functions of regulating ccRCC, such as ANKHD1 and QKI 18, 19.

In this study, we used the high-throughput expression profiles (HTSeq-counts) to explore the prognostic significance of RNA Binding Proteins (RBPs) in ccRCC. There are 200 differently expressed RBPs (DERBPs) in ccRCC. The biological functions of these DERBPs were analyzed by GO and KEGG enrichment analyses, and biological hub genes were explored by the PPI network. Furthermore, univariate Cox regression and LASSO analysis showed that the expression of 10 DERBPs was associated with overall survival (OS) in the training cohort. Afterward, a 10-DERBPs-based prognostic signature was built and validated. Then patients in the TCGA database were divided into low-risk and high-risk groups. The effect of the prognostic signature and its clinical significance was tested.

## Materials and Methods

### Extraction of Clinical and gene expression data

The list of 1912 RBPs was obtained according to the previous study. The transcriptome profiling (HTSeq-counts) of 611 samples, including 539 ccRCC samples and 72 normal kidney samples, were downloaded from the TCGA database (http://portal.gdc.cancer.gov/). Next, gene annotation was performed using the Ensemble database. Meanwhile, the clinical data were obtained from the XENA database (xenabrowser.net/heatmap/). The whole samples were considered as the training cohort, and half of the samples were randomly selected as the internal validation cohort (n=265). Moreover, the expression data and corresponding clinical data of the external validation cohort were obtained from the GEO database (GSE29609, n=39). Meanwhile, the mRNA expression and the corresponding clinical information of the other external validation cohort were downloaded from the ICGC database (https://icgc.org/, n=91). Entrez Gene IDs and R studio (version 3.6.1) were used for subsequent analyses in the cohorts above.

### Identification of DERBPs

"EdgeR" package was used to normalize the count matrix and identify DERBPs. Those RBPs with | Log2Foldchange| >1 and False discovery rate (FDR)<0.05 were considered to have statistical significance.

### Functional and Pathway Enrichment

To explore the potential biological functions of DERBPs, we performed GO and KEGG enrichment analyses using R packages such as "clusterProfiler," "enrichplot," "org.Hs.eg.db," and "ggplot2". Moreover, the GO enrichment included biological processes (BP), molecular functions (MF), and cellular component (CC). P-value<0.05 was considered statistically significant 20.

### Protein-protein interaction (PPI) network

In this study, a protein-protein interaction network of DERBPs was constructed by STRING (version 11.0). Then Cytoscape 3.7.1 was used to analyze and visualize the network. Genes with degrees≥20 were screened as biological hub genes, and interactions with a combined score of>0.4 were considered significant. MCODE, a plugin for Cytoscape, was used to identify the most significant module with selections as MCODE score>4 and nodes>4.

#### Identification of prognostic DERBPs

Kaplan-Meier survival analyses were performed to evaluate the effects of each RBPs for the OS of ccRCC patients. We screened out the prognostic RBPs by using univariate Cox regression and choosing prognostic RBPs with p<0.05. The DERBPs with | Log2Foldchange | >1 and FDR < 0.05 were retained and intersected with prognostic RBPs with p<0.05. The overlapping genes from two sets were used as prognostic DERBPs for the subsequent analysis.

### Construction and validation of the prognostic signature

The patients with survival time less than 30 days were excluded. Then, we randomly split the samples from TCGA into the training group (n=359) and the validation group (n=154) at a 7:3 ratio. Hereafter, using "glmnet" packages, LASSO Cox regression analysis was applied at 1000 maxit cross-validation to construct the signatures 21. Besides, we defined the formula for calculating the prognostic risk score as follow: Risk score = coef(gene1)*Exp(gene1) + coef(gene2)* Exp (gene 2) + … + coef(genen)* Exp (genen). Where "coef" represented the coefficient score estimated by multivariate Cox analysis, and "Exp" represented the expression value of the individual gene. The risk score was used as a measure of prognostic risk for each ccRCC patients. Meanwhile, according to the formula established in the training cohort, we calculated the risk score of the samples in the validation cohorts, including TCGA internal, GEO external, and ICGC external validation cohorts. We then classified the ccRCC patients into high-risk and low-risk groups, according to the median risk score as the cut-off. Kaplan-Meier survival analyses and the time-dependent ROC curve were used to evaluate the predictive performance of the prognostic signature. The AUC value of 0.75 or higher was considered a significant predictive value, and the value of 0.60 or higher was regarded as acceptable for prediction.

### Statistical analysis

Statistical analyses were performed by using R studio (version 3.6.1) and SPSS (version 26.0). Kaplan-Meier survival analyses were used to estimate the survival rate. The time-dependent ROC curve was used to evaluate the accuracy of the prognostic signature. Besides, the Cox regression model was used to explore the independent risk factors for the OS in univariate and multivariate analyses. The samples with survival time less than 30 days, NX, MX, or missing values were excluded. Then a nomogram was developed by using the Cox regression model and made by the 'RMS' package. Concordance index and calibration plot were used to estimate the performance of the nomogram. All p-values are based on a two-sided statistical analysis, and p < 0.05 was considered to indicate statistical significance.

## Results

### Identification of DERBPs between tumor and normal in ccRCC tissue

**Figure 1:**
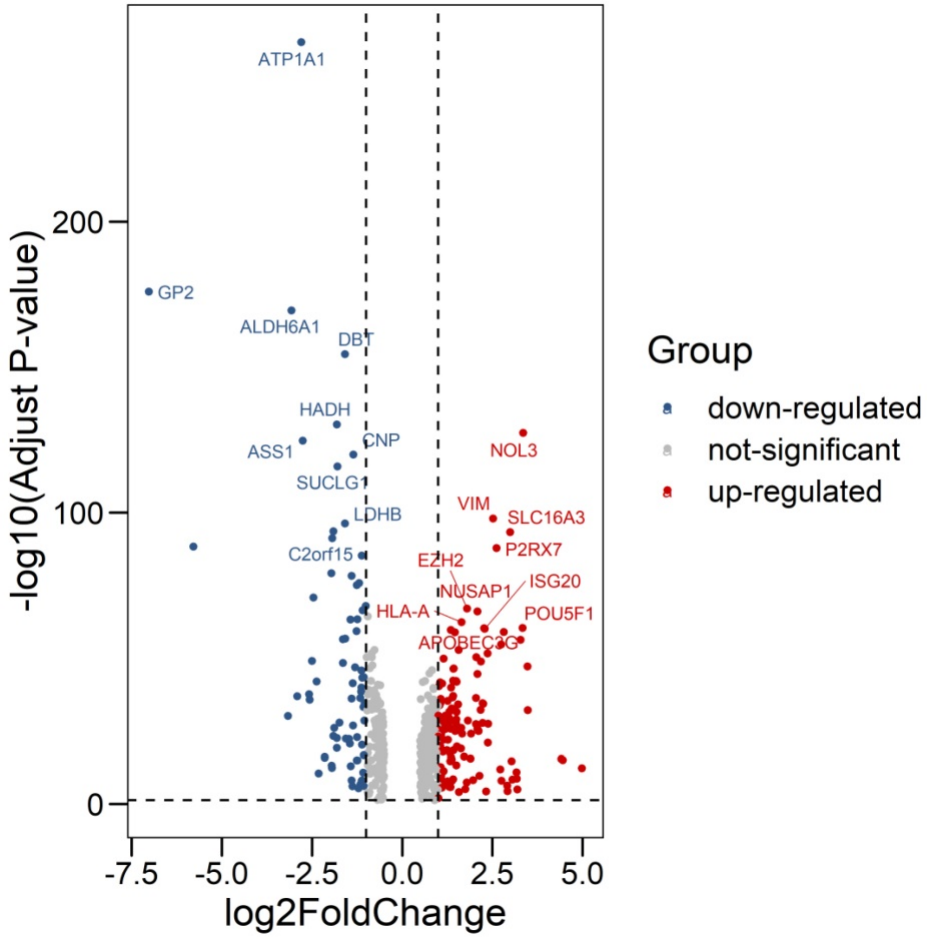
Differentially expression of RNA Binding Proteins in ccRCC tissue samples. The differential expression of 200 RBPs was shown in the -log (Adjust-P-value) vs. log (Fold-Change) plot. Top 10 most significant RBPs in up-regulated and down-regulated groups were labeled with gene symbol ID.

**Figure 2:**
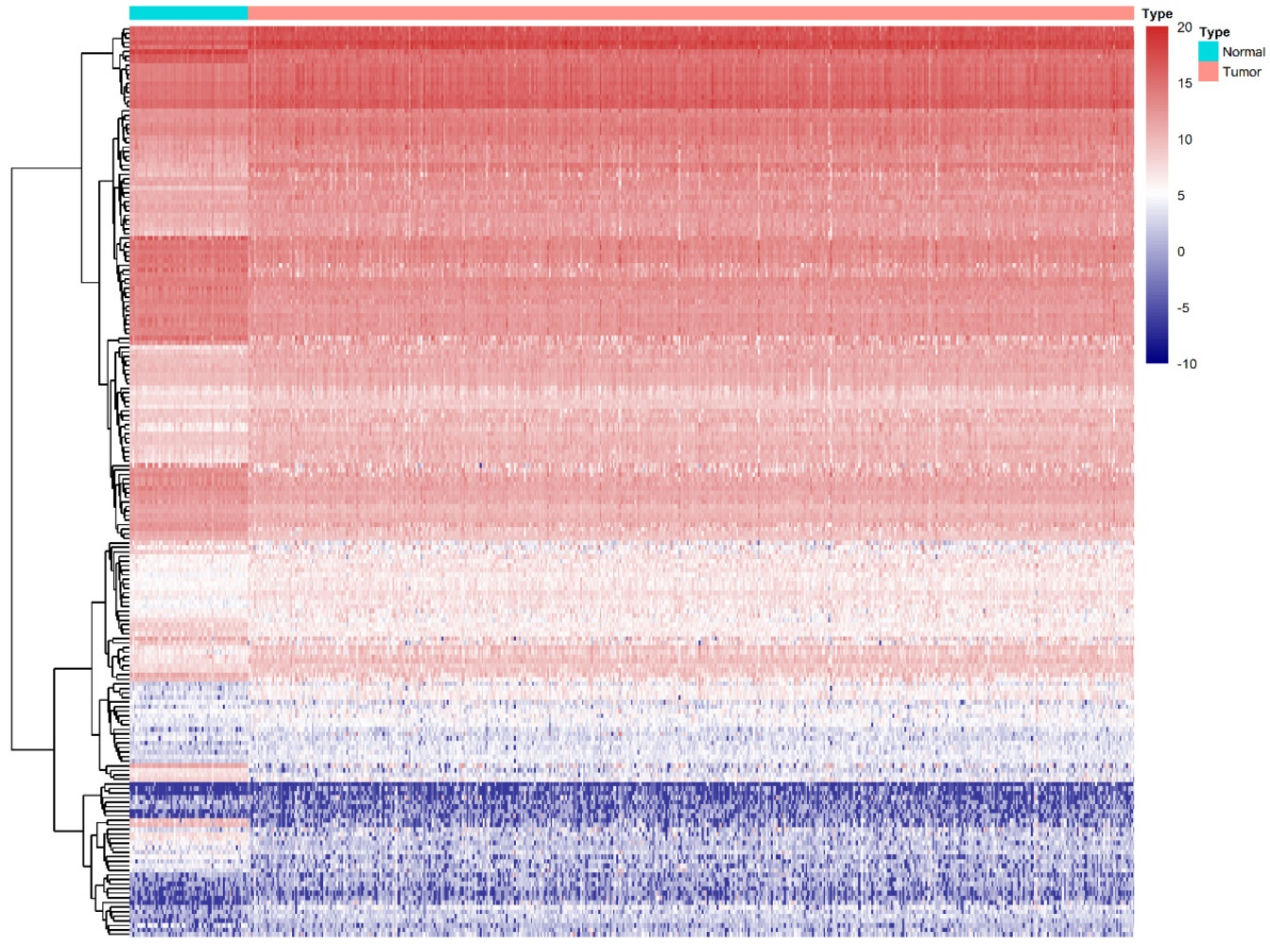
Differentially expression of RBPs in ccRCC. Unsupervised clustering analysis was performed using the R package "Pheatmap," based on log2-transformed count values. The columns are samples, and the rows are RBPs. The blue represents down-regulation, while the red represents up-regulation.

We compared all the mRNA expression levels of RBPs between 72 normal kidney tissue and 539 kidney renal clear cell carcinoma (KIRC) tissue in the TCGA-KIRC dataset. We found that a total of 200 differentially expressed RBPs (FDR<0.05, |log2FoldChange|>1), including 128 up-regulated and 72 down-regulated RBPs (Table S1). Moreover, the 10 most significant DERBPs in both up-regulated and down-regulated groups were shown (Figure 1). By using the "pheatmap" R package, an expression heatmap for all DERBPs was constructed either (Figure 2).

### Bioinformatics analysis of DERBPs in ccRCC

**Figure 3:**
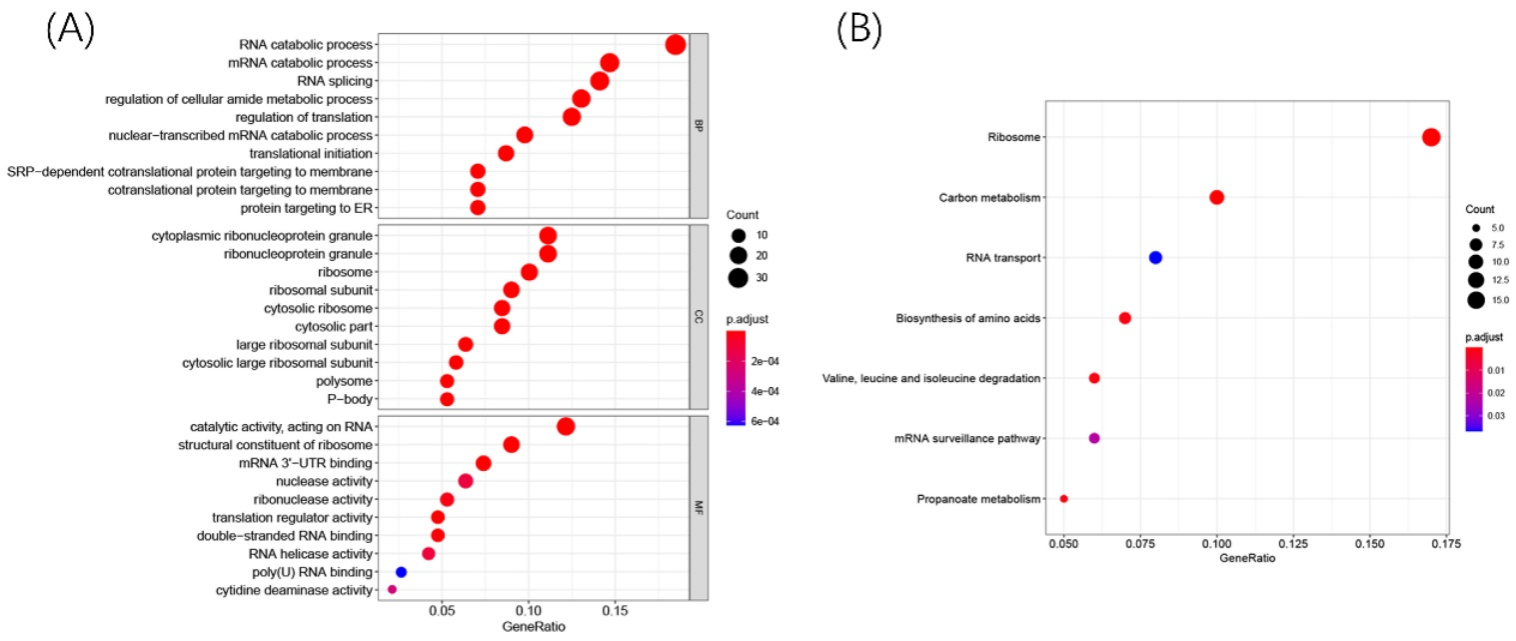
R packages were used to explore GO and KEGG enrichment pathways among DERBPs. Dot plots for GO (A) and KEGG (B) enrichment pathways were shown. The size of the dot represents counts, and the color of the dot represents p-value.

To explore the potential biological function and mechanisms of DERBPs in regulating ccRCC, we performed KEGG and GO enrichment analyses. The GO results showed that these DERBPs could participate in some vital biological processes such as mRNA processing, RNA splicing, and DNA modification (Figure 3A). Moreover, KEGG pathway enrichment analysis showed that DERBPs were mainly involved in Ribosome, Carbon metabolism, and RNA transport (Figure 3B).

**Figure 4:**
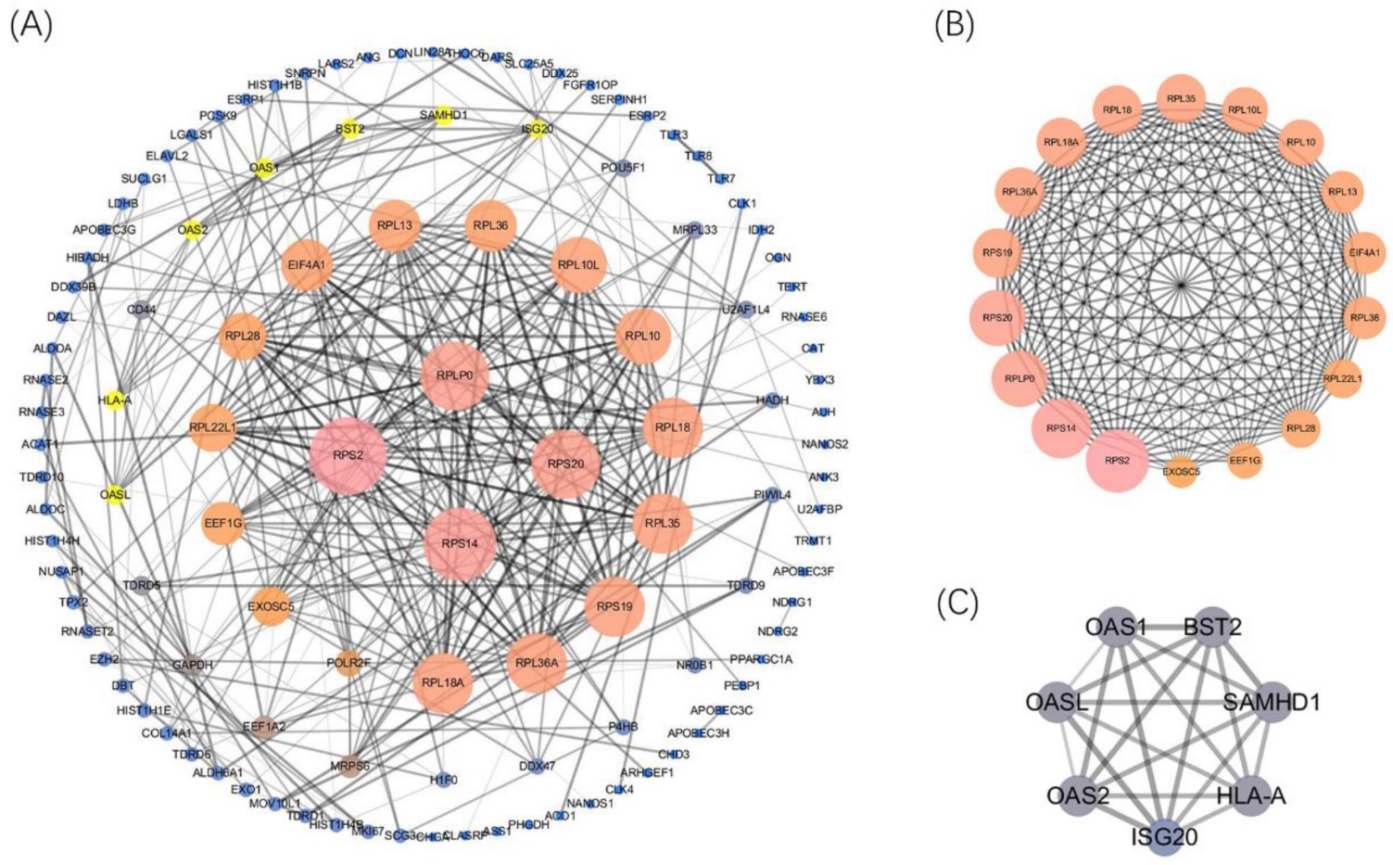
Construction of the PPI network and 4 hub genes were identified(A). Selected important modules of target genes with MCODE score≥4, nodes≥4(B, C).

Afterward, the PPI network was built (confidence=0.7) by using STRING 11.0 better to explore the roles of these DERBPs in regulating ccRCC. We then used Cytoscape to analyze and construct a PPI network, including 117 nodes and 328 edges, where low degree value is correlated with small node size, and low co-expression value is related to small edge size. According to the degree value, 4 hub RBPs were found with degree>20 (Figure 4A). This co-expression result showed that RPS2, RPS14, RPS20, and RPLP0 might play essential roles in ccRCC. The detailed information of 4 biological hub genes was shown (Table S2). Then, the MCODE plugin was used to explore the critical modules of target genes, and two imperative modules were selected (Figure 4B,4C).

### Biological pathway enrichments and prognostic value of the biological hub genes

**Figure 5:**
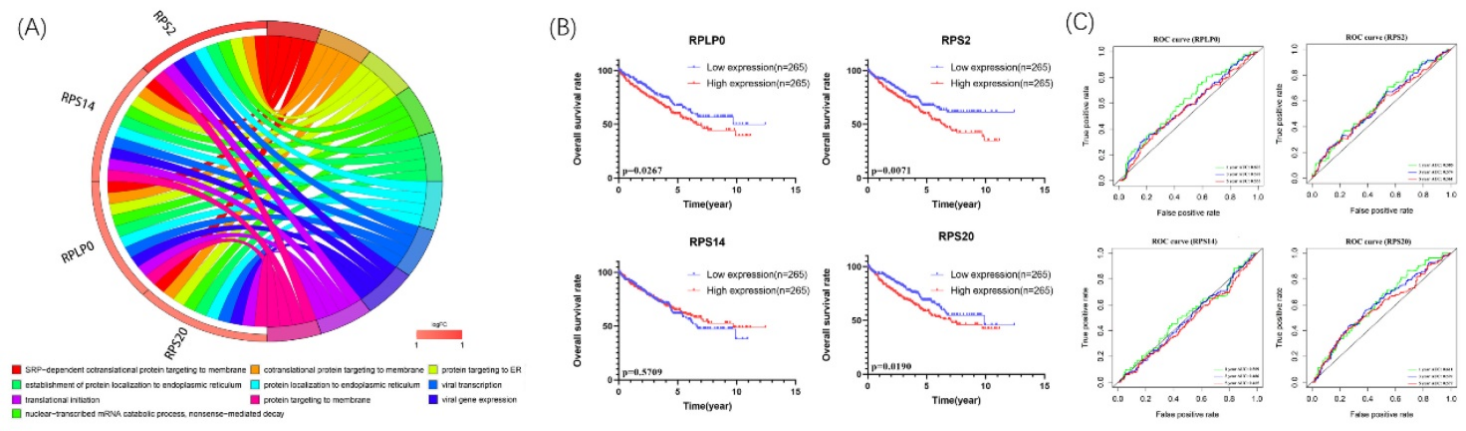
Circle plots for 10 most significant GO enrichment of 4 hub genes were shown (A). The size of the dot represents counts, and the color of the dot represents p-value. Moreover, Kaplan-Meier survival and time-dependent ROC curve analyses of 4 hub genes were shown (B,C).

We further explore the potential biological pathways of 4 biological hub genes. The GO analyses indicated that 4 biological hub genes were involved in some vital biological processes such as protein targeting, RNA binding, and ribosomal subunit (Figure 5A). Besides, there were just 1 KEGG pathways enriched, which is Ribosome. Then, according to the median expression of the hub genes, the patients in the TCGA database were divided into high-risk (n=265) and low-risk (n=265) groups. We performed Kaplan-Meier survival analyses to examine the survival significance between high- and low-risk groups. Patients with high expression of RPLP0 (p=0.0267), RPS2 (p=0.0071), RPS20 (p=0.0190) were significantly related to the poor prognosis (Figure 5B). Then we used ROC curves to evaluate the prognostic performance of the hub genes. The outcome indicated that none of the 4 hub genes could accurately predict the 1-year, 3-year, and 5-year prognosis of ccRCC patients (Figure 5C).

#### Identification of prognostic DERBPs

**Figure 6:**
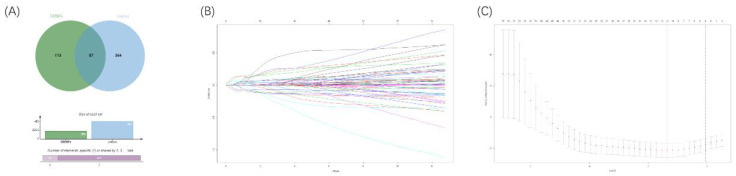
Venn diagram of overlapping prognostic DERBPs from prognostic RPBs (OS univariate cox p<0.05) and DEMRGs (|logFC| >1 and padj < 0.05) (A). 1000 maxit cross-validation for tuning parameter selection in the LASSO model for OS (B). LASSO coefficient profiles of prognostic DERBPs for OS (C).

To explore an optimal model for predicting the prognosis of ccRCC patients, we continued to implement univariable Cox regression analysis with the p-value of <0.05 (n=451). Following this, the overlapped RBPs in both prognostic RBPs and DERBPs were selected for subsequent analyses (Figure 6A). Then, the LASSO Cox regression analysis was performed in the training cohort at 1000 maxit (Figure 6B, 6C), and we identified 10 DERBPs (ANK3, CD44, CGN, CHGA, DQX1, IGF2BP2, IGF2BP3, PABPC1L, KIAA1324, and RPL22L1) as potential risk genes in OS prognostic signature. Furthermore, the detailed information of 10 DERBPs was listed in the table (Table 1).

### Construction and validation of the prognostic signature

**Figure 7:**
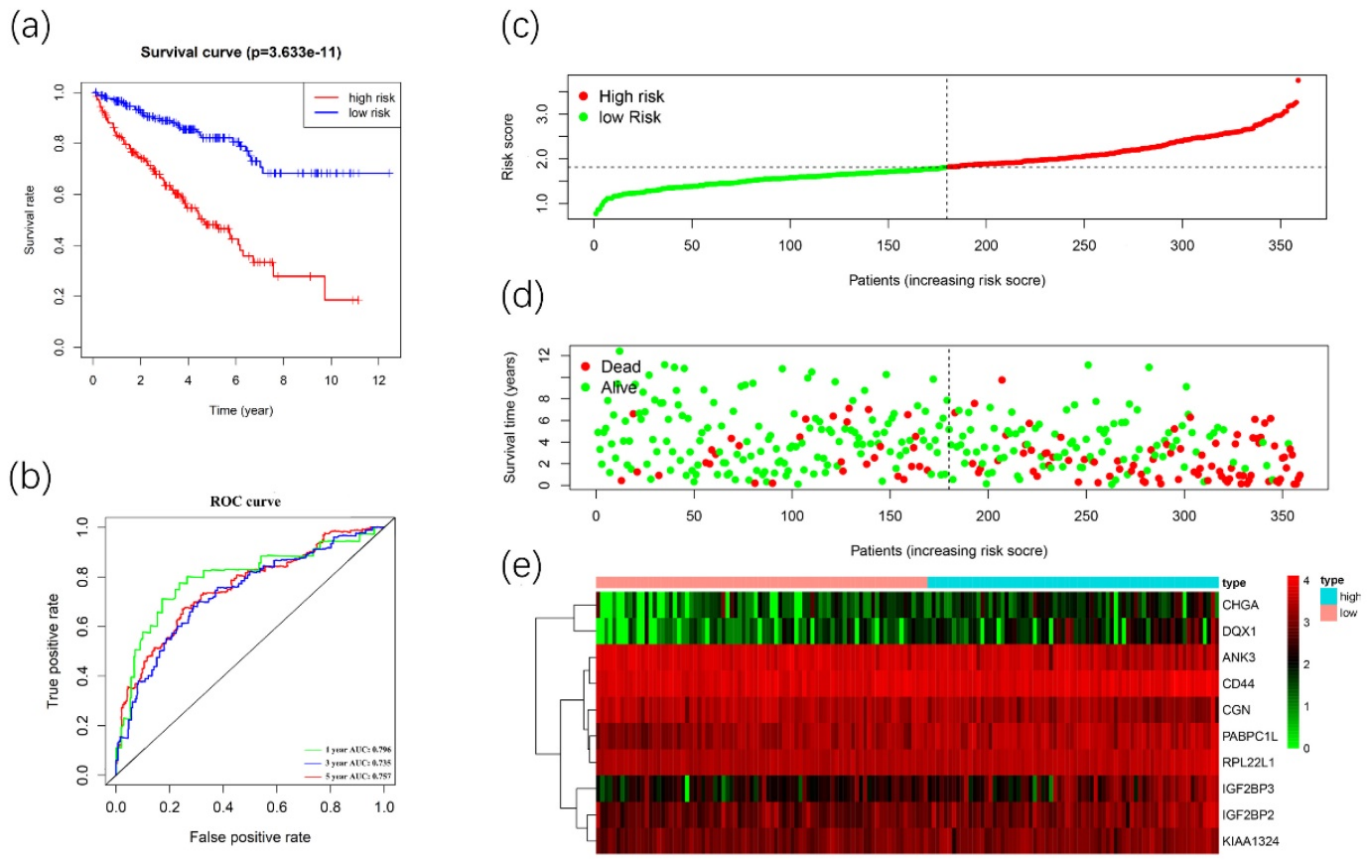
Construction of the prognostic signature. Analysis of OS prognostic signatures in ccRCC patients. Kaplan-Meier survival curve of OS in high-risk (red line) and low-risk (blue line) in ccRCC patients(A). Time-dependent ROC curve show area under curve (AUC) values at 1years(green), 3 years (blue) and 5 years (red) in ccRCC(B). Risk score distribution of high-risk (red) and low-risk (green) ccRCC patients in the OS model(C). A Scatter plot shows the survival status of ccRCC patients in the OS model. Red dots denote patients that are dead, and green dots denote patients that are alive(D). Expression of risk genes in the high-risk (blue) and low-risk (pink) training group ccRCC patients in the OS model(E).

We used these 10 DERBPs above to construct a prognostic signature for predicting the OS of ccRCC patients. Then, according to the median risk score, the patients in the training cohort were divided into high-risk (n=179) and low-risk (n=180) groups for OS. The high-risk group has a worse prognosis than the low-risk group by using Kaplan-Meier survival analyses (Figure 7A). We found that the 1-year, 3-year, and 5-year survival rate in the high-risk group were 84.2%, 64.5%, and 46.9%, while the 1-year, 3-year and 5-year survival rate in the low-risk group were 96.2%, 87.8%, and 80.7%. Then we used time-dependent ROC curves to predict the performance of the prognostic signature. The 1-year, 3-year, and 5-year AUC values were 0.784, 0.742, and 0.771 (Figure 7B). Furthermore, the distribution of risk score, survival status, and the heatmap of risk RBPs were shown (Figure 7C-7E).

**Figure 8:**
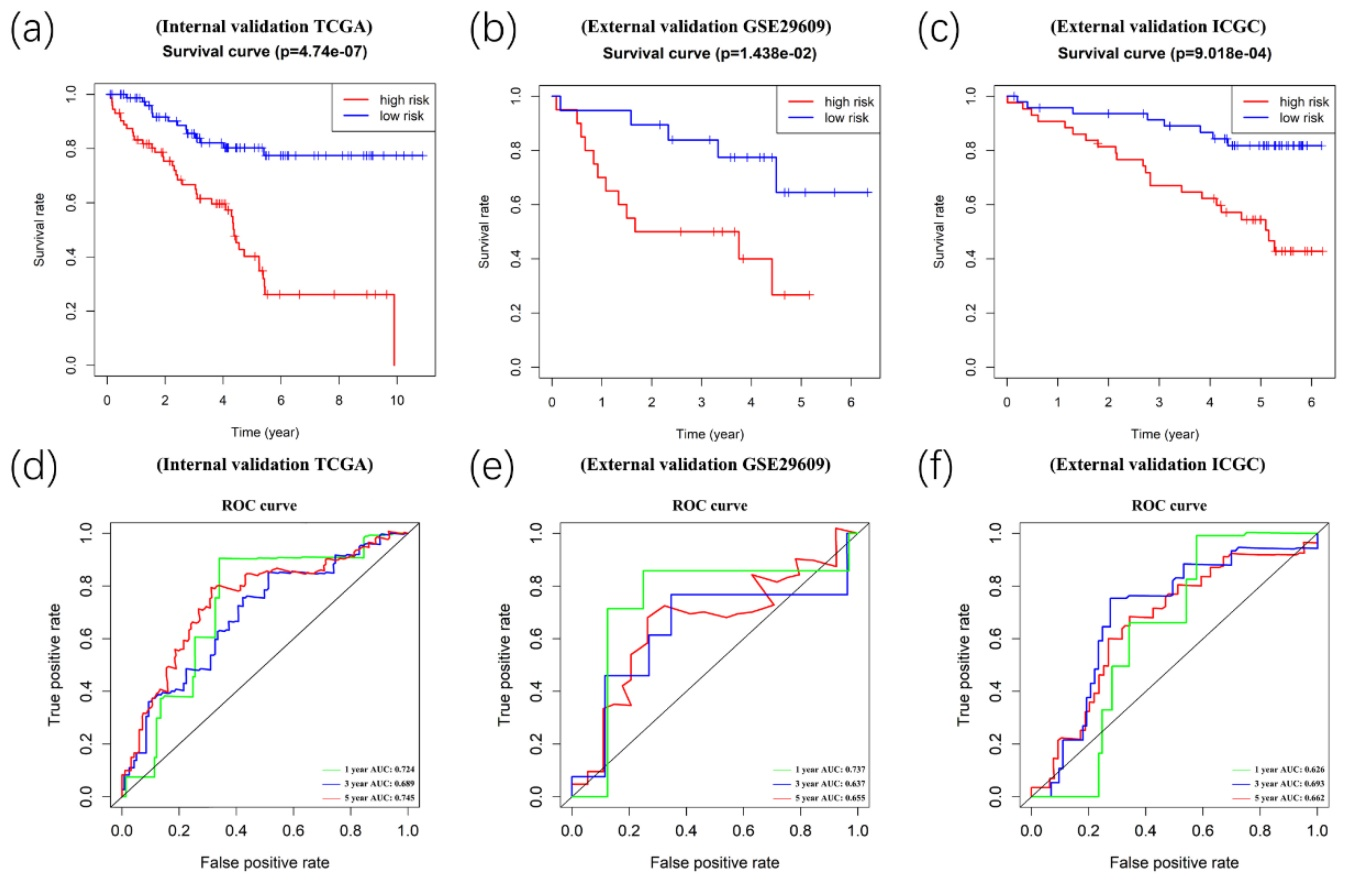
Validation of the prognostic signature. Kaplan-Meier survival analyses showed significant differences between the high- (red line) and low-risk groups (blue line) in the TCGA internal(A), GEO external(B), and ICGC external (C) validation cohorts. Meanwhile, time-dependent ROC curve analyses were performed at 1-year, 3-year, and 5-year OS of ccRCC patients in the TCGA internal (D), GEO external (E), and ICGC external (F) validation cohorts

Afterward, we validated the accuracy of the prognostic signature in the internal and external validation cohorts. Following this, we calculated the risk score of each patient based on the expression of the risk genes. The patients in the validation cohort were divided into high- and low-risk groups. Kaplan-Meier survival analyses showed significant differences between the high- and low-risk groups (p<0.05) in the TCGA internal (n=154), GEO external (n=39), and ICGC external (n=) validation cohorts (Figure 8A-8C). Meanwhile, time-dependent ROC curve analyses were performed at 1-year, 3-year, and 5-year OS of ccRCC patients in the TCGA internal, GEO external, and ICGC external validation cohorts (Figure 8D-8F).

### The prognostic signature is independently associated with OS of ccRCC patients

Then, we analyzed the correlation between the prognostic signature and clinical parameters of ccRCC patients. The information of 530 ccRCC patients in the TCGA database was shown (Table 2). Cox regression analysis was conducted by using the TCGA database, including signature, age, gender, grade, stage, pathologic T, pathologic M, and pathologic N. As it is shown, in univariate analysis, risk, grade, pT, pM, pN, and stage were significantly correlated with OS of ccRCC patients (Table 3, p<0.05). Meanwhile, multivariate analysis showed that age, pT, pM, and risk were significantly related to OS (Table 3, p<0.05). The outcome indicated that the prognostic signature could be independently used to predict OS in ccCC patients.

**Figure 9:**
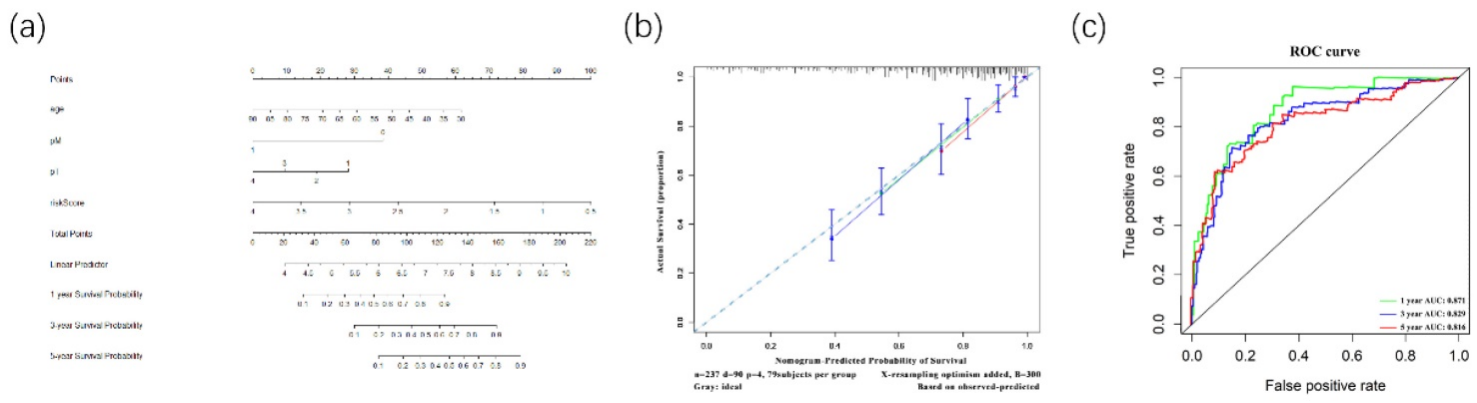
The Nomogram was constructed for OS(A). Its calibration curve showed that 1-year(red), 3-year(green) and 5-year(blue) effects of prediction(B). Meanwhile, the ROC curve showed the 1-year(red), 3-year(green), and 5-year(blue) OS prediction of the nomogram (C).

Furthermore, to better predict prognosis at 1-year, 3-year, and 5-year survival of ccRCC patients, we constructed a nomogram integrating riskscore and clinical parameters that have significance with OS in multivariate analysis (Figure 9A). Moreover, we used a calibration curve to assess the accuracy of the nomogram (Figure 9B). The AUC values for 1-year, 3-year, and 5-year OS were 0.871, 0.829, 0.816 (Figure 9C). Those data showed that the nomogram could accurately predict 1-year, 3-year, and 5-year OS of ccRCC patients.

## Discussion

Clear cell Renal cell carcinoma, especially metastatic ccRCC, is associated with high morbidity and mortality 22. Although some therapeutic targets have been found in recent years, such as VEGF and mTOR, the outcome of treatment was varied, and the majority of ccRCC patients eventually got poor prognosis 23. Besides, almost 25%-30% of ccRCC patients are found metastasis at initial diagnosis. Hence, finding diagnostic biomarkers or therapeutic targets for ccRCC is still meaningful. To our best knowledge, post-transcriptional regulation has been shown a correlation with tumorigenesis, such as RNA splicing and polyadenylation. Post-transcriptional regulation also means potential therapeutic opportunities 24. RNA Binding Proteins (RBPs) play important roles in all processes of post-transcriptional regulation, which significantly regulate the expression and function of oncoproteins and tumor suppressor proteins 25. However, the relationship between RBPs and ccRCC is still unclear.

In this study, we integrated high-throughput transcriptome profiling from the TCGA database and identified 200 DERBPs, including 128 up-regulated RBPs and 72 down-regulated RBPs. Furthermore, we analyzed biological pathways by using GO&KEGG and constructed a protein-protein interaction network. Firstly, for the biological process, DERBPs mainly occurred in mRNA processing, RNA splicing, translation, and location. Recent studies showed that RBPs could promote the malignant phenotypes of various cancers by regulating the mRNA process 26-28, consistent with DERBPs biological process. Secondly, for molecular functions, DERBPs could influence the binding of mRNA, mRNA 3'-UTR, and double-stranded RNA. Meanwhile, numerous activities of the transcriptional process were regulated by DERBPs. Next, the cell components showed that DERBPs were mainly located in the Ribosome. Dysregulation of ribosome biogenesis played a vital role in the development and progression of most spontaneous cancers 29. Moreover, KEGG showed that DERBPs were involved in metabolism and transcriptional processes such as carbon metabolism and RNA transport.

Last but not least, by constructing a PPI network, we found that 4 biological hub genes with degree>20, including RPLP0, RPS2, RPS14, RPS20. The hub genes played critical roles in regulating the processes of tumorigenesis and progression of ccRCC. However, the ROC curve indicated that all the hub genes lacked the prognostic value. Additionally, all hub genes were associated with ribosomal protein, which corroborated GO&KEGG results.

Then we used univariable and LASSO Cox regressions and established a prognostic signature based on the 10 DERPBs to predict the prognosis of ccRCC patients. The outcome showed that the prognostic signature of 10 DERBPs could provide an accurate prognosis of ccRCC patients. The outcome indicated that the prognostic signature could be independently used to predict OS in ccCC patients. As shown in Table 3, in univariate analysis, risk, grade, stage, pT, pM, and pN were correlated with OS of ccRCC patients. Meanwhile, multivariate analysis showed that age, pT, pM, and risk were related to OS. Combined with significant clinical parameters and risk score, the nomogram was built to predict 1-year, 3-year, and 5-year survival probability. Moreover, the calibration curve was performed with 300 bootstraps resamples and showed that the prognostic signature conducted with the ideal model 30. Besides, the time-dependent ROC curve showed the accuracy of the nomogram.

Our study has demonstrated that the 10-DERBPs-based signature was strongly correlated with the overall survival of ccRCC patients. Some of the RBPs in our signature have been explored in the previous studies. CD44 was implicated in poor prognosis, cancer cell invasion, metastasis, and resistance to the sunitinib treatment 31. It could be regulated by NF-kB inhibitors, which influenced the cancer stem-like cells 32. IGF2BP3 were shown a strong association with the survival of ccRCC, which could activate the NF-kB pathway and promote RCC progression 33, 34. These genes should be further explored in the future, especially concerning ccRCC.

The major limitation of this study was that all data were obtained from several public databases, without the validation of prospective clinical trials. Moreover, some important clinical information, such as the treatment of ccRCC patients, was not available in the TCGA database. Besides, the mechanism of RBPs in the prognostic signature required detailed examinations in the future. Despite these limitations, our results showed that the prognostic signature based on RBPs could be a reliable predictive tool of ccRCC survival.

## Conclusion

In our current study, we explored the biological functions and prognostic value of RBPs in ccRCC. Bioinformatics analysis showed that DERBPs might regulate transcriptional processes to influence tumorigenesis and progression. Furthermore, the prognostic signature of DERBPs might serve as promising diagnostic and prognostic biomarkers in ccRCC. More studies should be performed to confirm the findings of our studies.

## Supplementary Material

Supplementary table.

## Figures and Tables

**Table 1:** Detailed information on DERBPs for constructing the prognostic signature.

A 10-DERBPs-based signature for OS				
Gene name	ENSG_ID	Chromosome	Description	Coef
ANK3	ENSG00000151150	10q21.2	Ankyrin 3	-0.081289
CD44	ENSG00000026508	11p13	CD44 Molecule	0.026241
CGN	ENSG00000143375	1q21.3	Cingulin	-0.136891
CHGA	ENSG00000100604	14q32.12	Chromogranin A	0.028761
DQX1	ENSG00000144045	2p13.1	DEAQ-Box RNA Dependent ATPase 1	0.006470
IGF2BP2	ENSG00000073792	3q27.2	Insulin-Like Growth Factor 2 MRNA Binding Protein 2	0.016286
IGF2BP3	ENSG00000136231	7p15.3	Insulin-Like Growth Factor 2 MRNA Binding Protein 3	0.065426
PABPC1L	ENSG00000101104	20q13.12	Poly(A) Binding Protein Cytoplasmic 1 Like	0.113194
KIAA1324	ENSG00000116299	1p13.3	Estrogen-Induced Gene 121 Protein	0.015845
RPL22L1	ENSG00000163584	3q26.2	Ribosomal Protein L22 Like 1	0.128415

**Table 2:** Clinical information of 530 ccRCC patients

Clinical parameters	Variable	Total (530)	Percentages (%)
Age	≤60	263	49.62
	>60	267	50.38
Gender	Male	344	64.91
	Female	186	35.09
Pathological T	T1	271	51.13
	T2	69	13.02
	T3	179	33.77
	T4	11	2.08
Pathological M	M0	420	79.25
	M1	78	14.72
	MX	32	6.04
Pathological N	N0	239	45.09
	N1	16	3.02
	NX	275	51.89
AJCC stage	Stage I	265	50.00
	Stage II	57	10.75
	Stage III	123	23.21
	Stage IV	83	15.66
	Unkonwn	2	0.38
ISUP grade	G1	14	2.64
	G2	227	42.83
	G3	206	38.87
	G4	75	14.15
	GX	8	1.51
Survival status	Dead	173	32.64
	Alive	357	67.36

**Table 3:** Univariate and Multivariate analyses of ccRCC patients in the TCGA database

Variables	Univariate analysis		Multivariate analysis	
	HR (95%CI)	p-value	HR (95%CI)	p-value
Risk model (High vs Low)	3.516 (2.175,5.686)	<0.001	2.953 (1.796,4.854)	<0.001
Age (>60 vs. ≤60)	1.519 (0.992,2.327)	0.054	1.806 (1.175,2.776)	0.007
Gender (Male vs. Female)	0.734 (0.703,1.649)	0.939		
Grade (G3+G4 vs. G1+G2 )	2.618 (1.639,4.180)	<0.001		
pT (T3+T4 vs. T1+T2)	3.002 (1.965,4.587)	<0.001	1.799 (1.137,2.847)	0.012
pM (M1 vs. M0)	4.210 (2.716,6.524)	<0.001	3.043 (1.904,4.864)	<0.001
pN (N1 vs. N0)	3.103 (1.602,6.011)	0.001		
Stage (III+IV vs. I+II)	3.430 (2.220,5.346)	<0.001		

## Abbreviations

ccRCC: clear cell Renal Cell Carcinoma
TCGA: The Cancer Genome Altas
ICGC: International Cancer Genome Consortium
KIRC: kidney renal clear cell carcinoma
RBPs: RNA-Binding Proteins
GEO: Gene Expression Omnibus
DERBPs: Differentially Expressed RNA-Binding proteins
OS: Overall Survival
LASSO: Least Absolute Shrinkage and Selection Operator
FDR: False Discovery Rate
ROC: Receiver Operating Characteristic
GO: Gene Ontology
KEGG: Kyoto Encyclopedia of Genes and Geomes

## References

[1] Miller KD, Nogueira L, Mariotto AB, Rowland JH, Yabroff KR, Alfano CM (2019). Cancer treatment and survivorship statistics, 2019. CA Cancer J Clin.

[2] Siegel RL, Miller KD, Jemal A (2020). Cancer statistics, 2020. CA Cancer J Clin.

[3] Shingarev R, Jaimes EA (2017). Renal cell carcinoma: new insights and challenges for a clinician scientist. Am J Physiol Renal Physiol.

[4] Jonasch E, Gao J, Rathmell WK (2014). Renal cell carcinoma. Bmj.

[5] Oliver GR, Hart SN, Klee EW (2015). Bioinformatics for clinical next generation sequencing. Clin Chem.

[6] Comprehensive molecular characterization of clear cell renal cell carcinoma Nature. 2013; 499: 43-9.

[7] Xu WH, Shi SN, Xu Y, Wang J, Wang HK, Cao DL (2019). Prognostic implications of Aquaporin 9 expression in clear cell renal cell carcinoma. J Transl Med.

[8] Wan B, Huang Y, Liu B, Lu L, Lv C (2019). a promising biomarker in clear cell renal cell carcinoma. PeerJ.

[9] Gerstberger S, Hafner M, Tuschl T (2014). A census of human RNA-binding proteins. Nat Rev Genet.

[10] Lunde BM, Moore C, Varani G (2007). RNA-binding proteins: modular design for efficient function. Nat Rev Mol Cell Biol.

[11] Wang K, Li L, Fu L, Yuan Y, Dai H, Zhu T (2019). Integrated Bioinformatics Analysis the Function of RNA Binding Proteins (RBPs) and Their Prognostic Value in Breast Cancer. Front Pharmacol.

[12] Fu XD, Ares M (2014). Context-dependent control of alternative splicing by RNA-binding proteins. Nat Rev Genet.

[13] Moore MJ, Proudfoot NJ (2009). Pre-mRNA processing reaches back to transcription and ahead to translation. Cell.

[14] Sonenberg N, Hinnebusch AG (2009). Regulation of translation initiation in eukaryotes: mechanisms and biological targets. Cell.

[15] Sebestyén E, Singh B, Miñana B, Pagès A, Mateo F, Pujana MA (2016). Large-scale analysis of genome and transcriptome alterations in multiple tumors unveils novel cancer-relevant splicing networks. Genome Res.

[16] Lukong KE, Chang KW, Khandjian EW, Richard S (2008). RNA-binding proteins in human genetic disease. Trends Genet.

[17] Zhu Z, He A, Lv T, Xu C, Lin L, Lin J (2019). Overexpression of P4HB is correlated with poor prognosis in human clear cell renal cell carcinoma. Cancer Biomark.

[18] Fragiadaki M, Zeidler MP (2018). Ankyrin repeat and single KH domain 1 (ANKHD1) drives renal cancer cell proliferation via binding to and altering a subset of miRNAs. J Biol Chem.

[19] Zhang RL, Yang JP, Peng LX, Zheng LS, Xie P, Wang MY (2016). RNA-binding protein QKI-5 inhibits the proliferation of clear cell renal cell carcinoma via post-transcriptional stabilization of RASA1 mRNA. Cell Cycle.

[20] Yu G, Wang L-G, Han Y, He Q-Y (2012). clusterProfiler: an R package for comparing biological themes among gene clusters. OMICS.

[21] Engebretsen S, Bohlin J (2019). Statistical predictions with glmnet. Clin Epigenetics.

[22] Hsieh JJ, Purdue MP, Signoretti S, Swanton C, Albiges L, Schmidinger M (2017). Renal cell carcinoma. Nat Rev Dis Primers.

[23] Motzer RJ, Hutson TE, McCann L, Deen K, Choueiri TK (2014). Overall survival in renal-cell carcinoma with pazopanib versus sunitinib. N Engl J Med.

[24] Obeng EA, Stewart C, Abdel-Wahab O (2019). Altered RNA Processing in Cancer Pathogenesis and Therapy. Cancer Discov.

[25] Pereira B, Billaud M, Almeida R (2017). RNA-Binding Proteins in Cancer: Old Players and New Actors. Trends Cancer.

[26] Mukohyama J, Shimono Y, Minami H, Kakeji Y, Suzuki A (2017). Roles of microRNAs and RNA-Binding Proteins in the Regulation of Colorectal Cancer Stem Cells. Cancers (Basel).

[27] Sun X, Hu Y, Wu J, Shi L, Zhu L, Xi P-W (2018). RBMS2 inhibits the proliferation by stabilizing P21 mRNA in breast cancer. J Exp Clin Cancer Res.

[28] Hodson DJ, Screen M, Turner M (2019). RNA-binding proteins in hematopoiesis and hematological malignancy. Blood.

[29] Pelletier J, Thomas G, Volarević S (2018). Ribosome biogenesis in cancer: new players and therapeutic avenues. Nat Rev Cancer.

[30] Iasonos A, Schrag D, Raj GV, Panageas KS (2008). How to build and interpret a nomogram for cancer prognosis. J Clin Oncol.

[31] Mikami S, Mizuno R, Kosaka T, Saya H, Oya M, Okada Y (2015). Expression of TNF-α and CD44 is implicated in poor prognosis, cancer cell invasion, metastasis and resistance to the sunitinib treatment in clear cell renal cell carcinomas. Int J Cancer.

[32] Ma C, Komohara Y, Ohnishi K, Shimoji T, Kuwahara N, Sakumura Y (2016). Infiltration of tumor-associated macrophages is involved in CD44 expression in clear cell renal cell carcinoma. Cancer Sci.

[33] Tschirdewahn S, Panic A, Püllen L, Harke NN, Hadaschik B, Riesz P (2019). Circulating and tissue IMP3 levels are correlated with poor survival in renal cell carcinoma. Int J Cancer.

[34] Pei X, Li M, Zhan J, Yu Y, Wei X, Guan L (2015). Enhanced IMP3 Expression Activates NF-кB Pathway and Promotes Renal Cell Carcinoma Progression. PLoS ONE.

