# Supplementary material for: Biological functions and prognostic value of RNA Binding Proteins in clear cell Renal Cell Carcinoma
Source: J Cancer. 2020 Sep 23;11(22):6591–600. doi: 10.7150/jca.49175 (PMC7545679; doi:10.7150/jca.49175)
Supplement: Supplementary file 1 — Supplementary table. [file jcav11p6591s1.pdf]

Table S1 Compared with normal tissues, 200 DERBPs were identified including 128 up-regulated RBPs and 72 down-regulated RBPs

DEGs	RBPs
Up-regulated	RPS20,HIST1H1E,RPL10,RPLP0,DARS,RPS14,THOC6,RPL28,APOBEC3F,U2AF1L4,RPL35,ALDOA,NOP16,TDRD10,HIST1H4H,RPL18A,RPL13,EEF1G,CLK1,TRMT1,RPL18,PLA2G1B,QTRT1,SRRM3,ARHGEF1,NOVA2,CLASRP,MEX3B,ZC3HAV1L,STK10,AFF2,RPL36,RPL36A,PABPC4L,PIWIL4,EXOSC5,YBX2,BST2,OAS2,NDRG1,FGFR1OP,ELAVL2,RPL10L,MOV10L1,RBM44,RPL22L1,XIRP1,AEN,CD44,P4HB,LGALS3,SAMHD1,ELAVL3,OAS1,RPS19,CELF6,RPS2,GAPDH,AFF3,CLK4,YBX3,ZC3H12D,TLR3,RNASE6,FBXO17,DDX39B,U2AF1,SERPINH1,FSCN1,OASL,DDX47,ARL6IP4,IFI16,RIMS1,POLR2F,ELAVL4,EXO1,HLA-A,ANG,KIAA1324,EEF1A2,CELF5,EZH2,TDRD6,NXF5,RDM1,PIWIL3,EIF4A1,TLR7,APOBEC3C,LGALS1,TLR8,NUSAP1,PABPC1L,RNF113B,MKI67,MTCL1,TPX2,RNASE10,ALDOC,APOBEC3G,ISG20,CPLX2,CORO1A,RNASE3,RNASE2,VIM,P2RX7,DQX1,PATL2,NANOS2,APOBEC3H,DDX53,DAZ1,SLC16A3,IGF2BP3,C4BPA,PDIA2,HIST1H4B,NR0B1,RNASET2,POU5F1,NOL3,JAKMIP1,RBM46,SCG3,TERT,HIST1H1B,
Down-regulated	GP2,RALYL,ESRP1,ALDH6A1,TDRD5,ATP1A1,ASS1,DCN,RBM11,DDX25,CGN,KHDRBS2,LIN28A,CHGA,RBFOX1,ENOX1,DAZL,TDRD1,H1F0,XK,C2orf15,CELF3,AZGP1,HADH,MYH14,SUCLG1,PPARGC1A,MRPS6,NANOS1,DBT,LDHB,ESRP2,LAMA2,DSP,PHGDH,LARS2,ADAD2,CSDC2,CPEB3,CADM1,PCSK9,NDRG2,ANK3,CNP,CAT,APEH,COL14A1,SNRPN,ACAT1,NXF3,PEBP1,OGN,ADK,AUH,TDRD9,IDH2,ACO1,RBM47,SLC25A48,MRPL33,PIH1D3,TST,HIBADH,IPO13,NPM2,MSI2,CHD3,IGF2BP2,SLC25A5,CPEB4,SYNE1,KIF1C,

Table S2 The detailed information of 4 hub genes

Gene name	Gene ID	Protein name	Location	Expression status
RPS2	6187	Ribosomal Protein S2	16p13.3	Upregulated
RPS14	6208	Ribosomal Protein S14	5q33.1	Upregulated
RPS20	6224	Ribosomal Protein S20	8q12.1	Upregulated
RPLP0	6175	Ribosomal Protein Lateral Stalk Subunit P0	12q24.23	Upregulated
